# FAMILY HEALTH STRESS AND INSOMNIA SYMPTOMS AMONG OLDER ADULTS: THE MODERATING ROLE OF ACTIVITY ENGAGEMENT

**DOI:** 10.1093/geroni/igae098.2875

**Published:** 2024-12-31

**Authors:** Jeein Jang, Jeffrey Burr

**Affiliations:** University of Massachusetts Boston, Boston, Massachusetts, United States; University of Massachusetts Boston, Boston, Massachusetts, United States

## Abstract

Insomnia symptoms that arise from stress pose a significant physical and psychological health concern for older adults. Yet, little empirical research has explicitly examined the association between stress caused by family health concerns and insomnia symptoms, along with the role that activity engagement plays in moderating this relationship. Pearlin’s stress process model is used to frame this study. Using data from the 2018 and 2020 waves of the Health and Retirement Study (N=9,003), this study employed ordered logistic regression models to assess the link between family health stress and insomnia severity using four items (i.e., trouble falling asleep, nighttime awakening, early morning awakenings, and feelings of nonrestorative sleep). Additionally, the moderating role of overall activity engagement, as well as three activity domains (cognitive, social, and physical activities) were included in the analysis. All statistical analyses were adjusted for sociodemographic characteristics, health-related factors, and caregiver status. The results of the regression analysis show older adults experiencing ‘somewhat upsetting’ and ‘very upsetting’ family health stress had 23% and 49% higher odds, respectively, of experiencing more severe insomnia symptoms compared to those with no family health stress. In addition, the relationship was buffered by overall activity engagement between the ‘very upsetting’ group and insomnia symptoms in the logistic regression. For the specific activity domains, cognitive activities were the only ones that moderated the association between family health stress and insomnia symptoms. These findings offer implications for public health interventions that seek to promote activity engagement among older adults.

